# Evaluation of Reduced Single-Photon Emission Computed Tomography Imaging Protocols and Software Variability in 177Lu-DOTATATE Dosimetry: Protocol for an Exploratory Observational Trial

**DOI:** 10.2196/83248

**Published:** 2026-02-19

**Authors:** Takayuki Yagihashi, Kenta Miwa, Noriaki Miyaji, Satoru Sugimoto, Hideki Hayakawa, Noritoshi Kobayashi, Shoko Takano, Taro Murai

**Affiliations:** 1 Department of Medical Physics, Shonan Kamakura General Hospital, Kamakura, Japan; 2 Department of Radiological Sciences, Fukushima Medical University, Fukushima, Japan; 3 Medical Science Data-driven Mathematics Team, Division of Applied Mathematical Science, RIKEN Center for Interdisciplinary Theoretical and Mathematical Sciences, Yokohama, Japan; 4 Radiology Division, Shonan Kamakura General Hospital, Kamakura, Japan; 5 Department of Oncology, Yokohama City University Graduate School of Medicine, Yokohama, Japan; 6 Department of Radiation Oncology, Yokohama City University Graduate School of Medicine, Yokohama, Japan; 7 Department of Radiation Oncology, Shonan Kamakura General Hospital, 1370-1 Okamoto, Kamakura, 247-8533, Japan, 81 467-46-1717

**Keywords:** targeted radiopharmaceutical therapy, cancer, radioactivity, dosimetry, Lutetium-177

## Abstract

**Background:**

Targeted radiopharmaceutical therapy (TRT) offers a promising approach for cancer treatment by delivering radiation directly to tumor cells while sparing healthy tissues. Accurate dosimetry of organs and tumors is crucial to optimize therapeutic efficacy and minimize toxicity, particularly for dose-limiting organs such as the kidneys. Although routine dosimetry using single-photon emission computed tomography (SPECT) is recommended per guidelines, its widespread clinical application remains limited owing to a lack of consensus on the optimal frequency and timing of SPECT scans for accurate dosimetry, which leads to variability in clinical practice and hinders robust dose–response relationships. Furthermore, absorbed dose calculations rely on software-specific curve-fitting models. Thus, discrepancies in dose estimates among different simulation software programs pose a significant challenge to standardizing dosimetry workflows.

**Objective:**

We aimed to explore the optimal SPECT imaging schedule, evaluate differences among simulation software programs, and establish a standardized protocol for future epidemiological research to define organ tolerance doses and facilitate wider adoption of personalized TRT regimens.

**Methods:**

This exploratory observational trial will determine the optimal SPECT imaging protocol and consistency of dosimetry estimates using software programs for Lu-177-DOTATATE therapy in patients with neuroendocrine tumors. In a single treatment cycle, SPECT imaging will be performed at 4, 24, 96, and 168 hours following Lu-177-DOTATATE administration. The true absorbed dose cannot be directly measured; therefore, doses calculated using all time points (the 4-point method) for each patient will be used as the reference standard and compared with estimates derived from 1- to 3-point methods. Consequently, 15 dose simulations will be conducted per patient. This study implements a 5+5 design (rule-based design). The primary endpoint is the incidence of dose errors for each patient across different dose-calculation software systems. The kidney absorbed dose will be the primary focus of evaluation, with secondary analyses including tumor and other organ dosimetry. In principle, a dose error of <5% will be considered acceptable in each software system, depending on the absolute dose level. Differences among software systems and between patients will be evaluated using descriptive statistical methods. The study was approved by the Tokushukai Group Ethics Committee (Approval No. 2447).

**Results:**

Recruitment began on March 5, 2024, and 4 participants have been enrolled as of September 2025. Data collection is expected to be completed by February 2027, with the study results anticipated in August 2027.

**Conclusions:**

This study will evaluate whether simplified SPECT imaging protocols can maintain dosimetric accuracy while reducing the burden on patients and providers. It will also compare absorbed dose estimates across software programs to assess consistency and reliability as a reference. Findings will be disseminated through open-access peer-reviewed journals and relevant conferences and events.

## Introduction

Targeted radiopharmaceutical therapy (TRT) offers a promising cancer treatment approach by delivering radiation directly to tumor cells while minimizing exposure to surrounding healthy tissue. These radiopharmaceuticals, composed of radioactive isotopes linked to tumor-targeting molecules, are particularly effective for advanced cancers and are being explored for a wider range of malignancies [1-4]. Notable examples include Lutetium-177 (Lu-177)-DOTATATE for neuroendocrine tumors (NETs) and Lu-177-prostate-specific membrane antigen for metastatic castration-resistant prostate cancer [3,4]. Despite TRT’s expanding applicability, standardized methods for assessing absorbed doses and defining normal tissue tolerance thresholds remain underdeveloped.

Current guidelines from the European Council Directive 2013/59 (Article 56) and the European Association of Nuclear Medicine/Medical Internal Radiation Dose recommend routine assessment of absorbed radiation doses in each organ and tumor for patients undergoing TRT [5]. This dosimetry typically employs sequential quantitative single-photon emission computed tomography (SPECT) [5]. Accurate image quantification is crucial, as any underestimation or overestimation of absorbed doses can significantly affect clinical decisions and patient safety [5-7]. While SPECT images are routinely corrected for factors such as photon attenuation, scatter, and blurring to ensure quantitative accuracy [5-7], the widespread clinical application of standardized SPECT-based dosimetry remains limited.

Two primary challenges hinder the routine and standardized implementation of TRT dosimetry. First, there is no consensus on the optimal number or timing of SPECT scans. Accurate time–activity curves, which are essential for precise dose estimation, benefit from frequent imaging, particularly shortly after injection [8]. However, increasing SPECT scan frequency also raises patient burden, owing to additional radiation exposure, prolonged imaging time, and psychological stress, and strains health care resources. Consequently, current clinical practices for SPECT timing vary significantly, with protocols ranging from multiple scans (eg, 24, 96, and 168 hours post-injection) to a single scan at a specific time point [5,9-12]. This variability is particularly problematic for dose-limiting organs such as the kidneys, where precise dosimetry is critical to minimize toxicity. Second, absorbed dose calculations in TRT rely on various simulation algorithms, yet their dose estimates often lack universal validation. As a result, a dose reported by one software program may not be directly comparable with that from another, complicating the standardization of dosimetry workflows and hindering the establishment of robust dose–response relationships.

This study aims to identify the optimal SPECT imaging schedule for accurate TRT dosimetry and evaluate the differences in dose estimates among various simulation software programs. Ultimately, we seek to establish a standardized protocol for TRT dosimetry to support future epidemiological research, aid in defining organ tolerance doses, and facilitate the wider adoption of personalized TRT regimens, thereby optimizing therapeutic efficacy and minimizing toxicity.

## Methods

### Aims and Objectives

This study, conducted at Shonan Kamakura General Hospital, aims to optimize the SPECT imaging schedule, evaluate differences among simulation software programs, and develop a protocol suitable for epidemiological studies to define dose constraints for normal organs in Lu-177-DOTATATE therapy. Specifically, we will investigate whether the standard 4-time-point protocol could be reduced to 1-, 2-, or 3-scan protocols without compromising dosimetric accuracy. The purpose of this study is to explore practical time points for dosimetry within a single treatment cycle. Inter-cycle variability will be examined in subsequent studies after optimal time points are established in this study.

The primary endpoint is to evaluate the effect of scan reduction on the estimated absorbed dose to the kidneys, given that renal toxicity—particularly radiation-induced nephropathy—is the primary dose-limiting factor. Secondary endpoints include comparing tumor and normal organ dosimetry across imaging protocols and analyzing dose variations among software programs to assess consistency and reliability.

### Design

This open-label, single-institution exploratory observational study will recruit patients with neuroendocrine tumors undergoing Lu-177-DOTATATE therapy. Gamma emissions from normal organs and tumors will be measured using SPECT imaging at 4, 24, 96, and 168 hours following radiopharmaceutical administration. These images will serve as inputs for simulation software to calculate absorbed doses to normal organs and target tissues.

 This study adopts the 4-time-point SPECT protocol (4-point method) as the reference standard for each patient within each software program. Doses calculated after omitting 1‐3 time points (1-, 2-, and 3-point methods) will be compared with the 4-point dose within each program. The evaluation frameworks for each method are summarized below. Fifteen dose simulations will be conducted per patient. Data collection will span from March 2024 to February 2027.

For the 4-point method, dosimetry calculation will be performed using SPECT images taken at 4, 24, 96, and 168 hours. For the 3-point method, dosimetry calculation will be performed using SPECT images taken at 24, 96, and 168 hours; 4, 9, and 168 hours; 4, 24, and 168 hours; and 4, 24, and 96 hours. For the 2-point method, dosimetry calculation will be performed using SPECT images taken at 4 and 24 hours; 4 and 96 hours; 4 and 168 hours; 24 and 96 hours; 24 and 168 hours; and 96 and 168 hours. For the 1-point method, dosimetry calculation will be performed using SPECT images taken at 4 hours; 24 hours; 96 hours; and 168 hours post-injection

If dose discrepancies remain within acceptable limits, the simplified protocol will be considered for future trials. Additionally, absorbed doses calculated using each program will be compared across algorithms.

### Study Design Considerations

This exploratory observational study will evaluate various SPECT imaging time-point protocols and dosimetry software programs in patients undergoing Lu-177-DOTATATE therapy. Patient randomization is not applicable because the objective is to evaluate dosimetric methods against a reference standard in a real-world clinical setting rather than to compare treatment groups.

This is an open-label study. Blinding of participants or investigators will not be performed, as the primary focus is on technical comparisons of dosimetry methodologies and software programs. Blinding of imaging protocols or software analyses would be impractical and irrelevant for assessing dosimetric accuracy in this context.

### Safety and Data Monitoring

This study is designed as an exploratory observational trial and primarily involves the acquisition of additional SPECT/computed tomography (CT) images as part of the standard clinical protocol for Lu-177-DOTATATE therapy. As such, the research interventions are integral to routine clinical care and introduce minimal to no additional risks to participants beyond that associated with standard Lu-177-DOTATATE therapy.

The potential for additional radiation exposure from the extra SPECT/CT scans is considered negligible and will be kept as low as reasonably achievable, in accordance with the recommendations of the International Commission on Radiological Protection [13]. Any adverse events related to Lu-177-DOTATATE therapy will be managed and reported by the treating physicians according to standard clinical practice and existing institutional guidelines [14], and not as part of this study protocol, as the focus of this study is solely on dosimetry methodology.

Given the non-interventional and low-risk nature of this observational study, an independent data monitoring committee will not be established.

### Data Handling and Exclusions

All acquired SPECT/CT images that meet technical quality control standards will be included in the dosimetry analysis. Data points, including individual SPECT scans, may be excluded if deemed technically inadequate—owing to motion artifacts, hardware malfunction, or incomplete acquisition—after review by a medical physicist, as these issues could compromise dose calculation accuracy. All exclusions will be thoroughly documented and reported.

### Data Management

The data will be entered and stored using Microsoft Excel software, cross-checked in pairs, saved on an encrypted computer, and backed up.

### Statistical Analyses and Sample Size

**Figure 1.**
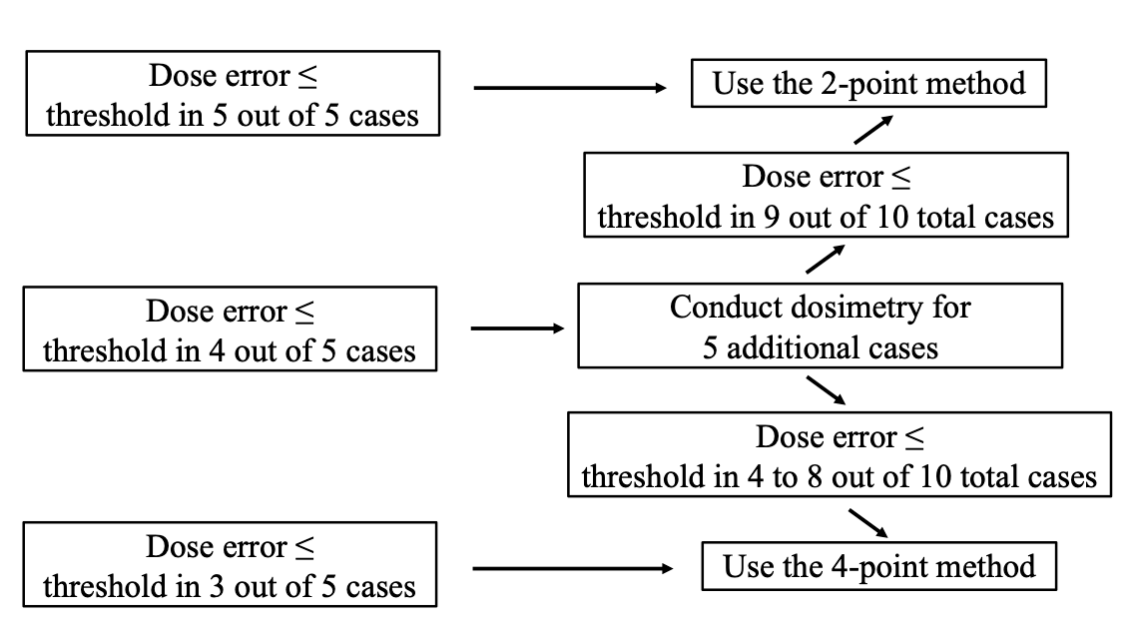
Workflow for stepwise evaluation of dose error acceptability. Dose errors are first evaluated in an initial set of five cases. If the dose error is within the predefined threshold in all five cases, the 2-point method is considered acceptable. If four out of five cases meet the criterion, the 4-point method is applied. If three or fewer cases meet the criterion, dosimetry is extended to five additional cases (total n = 10). Final acceptability is determined based on the number of cases within the threshold among all ten cases.

This study implements a 5+5 design, also known as a rule-based design [15]. The primary endpoint is the dose error incidence for each calculation algorithm. Figure 1 presents the decision-making workflow. The assumed dose error incidence in this study is set to be ≤5%; based on this assumption, the required number of eligible patients will be modified using the 5+5 design [16]. If the incidence (dose error per patient ≤ threshold) is ≤5% within the workflow, false-negative results will be observed in ≤23% of trials. If the incidence is ≥30%, false-positive results will occur in ≤17%.

Dose error below the predefined threshold will be considered acceptable for each calculation algorithm. The primary threshold will be set at 5%, following the International Commission on Radiation Units and Measurements Report 83 guidelines [17], which recommend that overall dosimetric uncertainty should ideally be within ±5%. For regions with absorbed doses <5 Gy, where larger errors are generally observed, the threshold will be relaxed to 10% [18]. A simplified method will be deemed “acceptable” if, in all five cases, the absorbed dose estimated by each software program falls within the threshold (5% or 10%, depending on the regional dose) when compared with the reference 4-point method. If multiple algorithms meet the criteria, priority will be given to the algorithm requiring the fewest time points for the subsequent trial. For example, if both the 3-point and 1-point methods meet the criteria, the 1-point method will be selected as the primary candidate. Among algorithms requiring the same number of time points, those incorporating the 4-hour or 24-hour sampling points will be preferred.

In external beam radiotherapy treatment planning, the total kidney dose threshold is 23 Gy [19], indicating that a dose of 5.5 Gy per treatment cycle is considered clinically acceptable. Therefore, we will relax the absorbed dose threshold from 5% to 10% if kidney doses are <5 Gy.

If the threshold is exceeded in 1 of the 5 patients, an additional 5 cases will be evaluated. The simplified method will be considered “acceptable” if at least 9 of the 10 cases meet the threshold. Conversely, if the dose error exceeds the threshold in 2 or more of the 10 cases, the simplified method will be considered “unacceptable.”

The true absorbed dose cannot be directly measured, and 4-point estimates are derived using software-specific kinetic models and dose-calculation frameworks. Therefore, it cannot be conclusively stated that these estimates are equivalent across different software programs. Consequently, consistency among software systems will be evaluated by comparing the magnitude and distribution of relative dose deviations from the 4-point reference and from the 1- to 3-point estimates using descriptive statistical methods. Visualization of these distributions, such as using box-and-whisker plots, may facilitate assessment of the validity of dose reduction at each time point. As a reference analysis, one-way analysis of variance will be used to compare absorbed doses to each organ of interest across software systems and between patients. The level of statistical significance is set at 0.05. If the *P* value is less than .05, the validity of the candidate calculation algorithm will be re-evaluated.

### Inclusion and Exclusion Criteria

The eligibility criteria are presented in Textbox 1 .

Textbox 1. **Eligibility criteria**

**Inclusion criteria**

- Age ≥20 years

- Blood tests within 1 month before treatment

- Estimated glomerular filtration rate >50 mL/min

- Hemoglobin level >10 g/dL

- White blood cell count between 3000 and 10,000/μL

- Platelet count >75,000/μL

- Albumin level >3 g/dL

- Bilirubin level <3 mg/dL

- Hasegawa dementia scale ≥21

- Ability to self-feed

- Ability to manage insulin and other medications

- Ability to manage urination (continence aids allowed)

- World Health Organization performance status score of 0 or 1

**Exclusion criteria**

- Urgent/critical findings on chest/abdominal computed tomography

- Hypersensitivity to Lu-177 DOTATATE, L-lysine HCl, or L-arginine HCl

- Pregnant/breastfeeding or women of childbearing potential without adequate contraception

- Male with partner of childbearing potential without effective contraception

- Psychiatric disorders impairing participation

- Systemic infection requiring ongoing treatment

- Unable to remain in a fixed position for >30 min

- Any condition deemed inappropriate by the principal investigator

### TRT Details

Lu-177-DOTATATE will be administered following the protocol previously approved by the Food and Drug Administration [20]. Based on this protocol, TRT will be administered in four treatment cycles per patient, with each cycle scheduled at 8-week intervals. In each cycle, Lu-177-DOTATATE (7.4 GBq) will be administered over 30 minutes. An amino acid solution containing 2.5% lysine and 2.5% arginine will be co-administered via intravenous infusion at approximately 1000 mL over 4 hours, beginning 30 minutes before TRT. This solution facilitates renal protection by promoting urinary excretion of Lu-177-DOTATATE, thereby reducing the dose absorbed by the kidneys. Prior to amino acid infusion, an appropriate antiemetic agent will be administered as an intravenous bolus to prevent nausea and vomiting.

### Dosimetry Methods

#### Dosimetry Protocol

In this study, four quantitative SPECT/CT scans will be performed for dosimetry following the administration of Lu-177-DOTATATE during one of the four treatment cycles. These scans, covering from the neck to the femoral head region, will be acquired over 30 minutes using a Symbia Intevo 6 system (Siemens Healthineers, Erlangen, Germany) equipped with medium-energy general-purpose collimators. To optimize image quality and quantitative accuracy, image reconstruction will be conducted using the ordered subset conjugate gradient minimization algorithm (xSPECT) with a matrix size of 256×256. A Gaussian filter will be applied during image reconstruction. The primary energy peak will be centered at 208 keV (187.6‐229.2 keV). Scatter correction will use the triple energy window method, and attenuation correction will be based on CT. CT images will be acquired at 130 kV and 20 mA using the adaptive dose modulation technique (CARE Dose 4D; Siemens Healthineers). To enable absolute quantification, the gamma camera will be calibrated using a Lu-177 point source. Dose calibration will be performed using serial SPECT/CT acquisitions of Lu-177 point sources in air and in a tissue-equivalent medium to simulate attenuation. Dead time will be determined and corrected based on wide-spectrum counting rates during image acquisition. To simulate absorbed doses in tumors and normal organs, the injected activity (GBq), date and time of TRT administration, and patient weight (kg) will be entered into the dosimetry software.

Subsequently, organ and target absorbed doses will be simulated using at least five commercially available software programs and one newly developed tool: (1) OLINDA/EXM (Hermes Medical Solutions); (2) Dosimetry Toolkit (GE HealthCare Medical Systems and Solutions); (3) Voxel Dosimetry (Hermes Medical Solutions); (4) MIM SurePlan MRT (MIM Software Inc.); (5) RT-PHITS (Japan Atomic Energy Agency); and (6) a newly developed dose calculation program introduced after 2022 (name and developer to be specified once the software is finalized and becomes available during the study period).

#### Quality Control and Assurance

To ensure the accuracy and reliability of dosimetry, this study will implement stringent quality control measures. Specifically, to evaluate inter-rater reliability in dose calculations, the following procedures will be carried out.

For all patient data, two independent radiation oncologists will each perform contouring of organs and tumors. If a discrepancy in contouring is observed between the two readings, the oncologists will discuss the findings and determine the final contours.

Dose calculations will be performed by an experienced and certified medical physicist. For quality assurance, a second experienced and certified medical physicist will independently verify the dose calculations.

### Recruitment

Participants will be enrolled after being administered at least one TRT cycle to minimize the risk of allergic reactions to the administered agents. Patients will not be enrolled until they have completed at least one treatment cycle and their ability to attend follow-up visits at our hospital has been confirmed. Eligible individuals will be invited to participate and will be provided with detailed study information by their radiation oncologist to support informed decision-making.

### Ethical Considerations

Ethics approval and consent to participate were reviewed and granted by the Tokushukai Group Ethics Committee Institutional Review Board (Approval No. 2447; UMIN000057478). Written informed consent will be obtained from all participants prior to study enrollment. All data will be de-identified prior to analysis, and no personally identifiable information will be accessible to the research team outside the treating institution. The study data will be handled in accordance with applicable data protection regulations. Participants will not receive any financial compensation for participation in this study. All study procedures will be conducted in accordance with the principles of the Declaration of Helsinki and applicable regulatory requirements.

## Results

Recruitment for this study began on March 5, 2024. As of September 2025, 4 participants have been enrolled. Data collection is expected to be completed by February 2027, and the study results are anticipated to be published by August 2027.

## Discussion

TRT is expected to become a standard treatment for various cancer types. While the prescribed dose should ideally be tailored to the tolerance of normal organs, standardized thresholds for normal tissue tolerance have not yet been fully established. Currently, no consensus exists on the optimal method for evaluating absorbed doses, including the appropriate number and timing of SPECT scans. As with external beam radiotherapy, tolerance thresholds for normal tissues should be validated through rigorous epidemiological studies [21]. Given potential regional differences in radiosensitivity, epidemiological data should be collected locally. At present, no prospective studies on Lu-177-DOTATATE dosimetry have been conducted in Japan—an unexpected gap, considering that fluorine-18 fluorodeoxyglucose positron emission tomography has already been optimized for the Japanese population [22].

A previous dosimetry study [23] has reported considerable variability in kidney dose estimates across institutions, with standard deviations as high as 58%. This high interfacility variability in absorbed kidney doses underscores the need for standardized workflows to reduce uncertainty in TRT dosimetry. Such variability impairs the ability to accurately assess and mitigate the risk of nephrotoxicity—a critical concern in TRT—and undermines the statistical power of large-scale epidemiological studies.

Therefore, ensuring the quality of SPECT, image acquisition protocols, and simulation software is essential for future epidemiological studies. Furthermore, workflows should be optimized to minimize the burden on patients and health care providers, as the feasibility of large-scale epidemiological studies depends on the participation of multiple facilities and patients. This protocol may be readily incorporated into routine clinical practice. In this study, we will obtain clinical data to determine whether simplified time points can achieve acceptable dosimetric accuracy. Absorbed doses in tumor tissue and normal organs across multiple calculation programs will be assessed using descriptive statistical methods.

Given the current absence of prospective Lu-177-DOTATATE dosimetry trials in Japan, the data generated from this study will be invaluable. It will serve as foundational epidemiological evidence crucial for establishing organ tolerance doses specifically for the Japanese population, thereby enhancing the safety and efficacy of TRT in the region. The findings are expected to offer new insights into Lu-177-DOTATATE dosimetry in neuroendocrine tumors and to support future epidemiological studies.

Regarding time-point selection, the maximum blood concentration of Lu-177-DOTATATE is observed within 1 hour. However, peak concentrations in normal organs and tumors occur thereafter. Within 4 hours post-administration, Lu-177-DOTATATE distributes to the kidneys, tumor lesions, liver, and spleen [24]. Although the early fast phase does not contribute substantially to the estimated renal absorbed dose, it can adversely affect the evaluation of the effective half-life. Therefore, it is generally accepted to acquire images after 4 hours and assume instantaneous uptake rather than to perform early imaging and risk misrepresenting the organ time activity–curve [25].

This study is exploratory and is intended to inform decision-making for future multi-institutional validation studies with larger cohorts. Thus, it has several intrinsic limitations. First, the findings may not be fully generalizable to other centers owing to variations in equipment and protocols, including software and imaging time points. The issue must be addressed through multi-institutional discussion prior to subsequent studies.

Second, the small sample size (estimated 5‐10 patients) may not capture the full range of pharmacokinetic variability or tumor responses among patients. However, given that 4-time-point measurements impose a substantial burden on patients and health care providers, the limited sample size is unavoidable from a practical standpoint. The study design allows for a false-positive probability of 32% if the incidence (dose error per patient≤ threshold) is ≤20% within the workflow. These dose errors are considered to arise from software-specific curve-fitting models and dose-calculation frameworks. The incidence of such errors will be addressed through cross-validation among multiple software systems. Specifically, simplified time-point methods that demonstrate inconsistencies between software systems will be excluded from consideration in subsequent studies. Third, detailed 4-time-point SPECT imaging is performed in only one treatment cycle per patient, which does not account for potential changes in radiopharmaceutical distribution across subsequent cycles. Inter-cycle variability will be examined in subsequent studies after the optimal time points are determined in the present study. Finally, although multiple simulation software programs are evaluated, not all possible variations in image acquisition parameters or software algorithms are explored. These limitations highlight the need for future multi-institutional validation studies with larger cohorts and longitudinal studies to comprehensively standardize TRT dosimetry. Despite these limitations, this study is expected to provide crucial foundational data to guide the development of robust dosimetry protocols essential for advancing personalized TRT and facilitating large-scale epidemiological research.

## Abbreviations

CT: computed tomography
SPECT: single-photon emission computed tomography
TRT: targeted radiopharmaceutical therapy

## References

[1] Sartor O, de Bono J, Chi KN (2021). Lutetium-177-PSMA-617 for metastatic castration-resistant prostate cancer. N Engl J Med.

[2] Strosberg J, El-Haddad G, Wolin E (2017). Phase 3 trial of 177Lu-Dotatate for midgut neuroendocrine tumors. N Engl J Med.

[3] Lapi SE, Scott PJH, Scott AM (2024). Recent advances and impending challenges for the radiopharmaceutical sciences in oncology. Lancet Oncol.

[4] Bela Andela S, Amthauer H, Furth C (2024). Quantitative PSMA-PET parameters in localized prostate cancer: prognostic and potential predictive value. Radiat Oncol.

[5] Ljungberg M, Celler A, Konijnenberg MW (2016). MIRD pamphlet no. 26: joint EANM/MIRD guidelines for quantitative 177Lu SPECT applied for dosimetry of radiopharmaceutical therapy. J Nucl Med.

[6] Sandström M, Garske U, Granberg D, Sundin A, Lundqvist H (2010). Individualized dosimetry in patients undergoing therapy with (177)Lu-DOTA-D-Phe (1)-Tyr (3)-octreotate. Eur J Nucl Med Mol Imaging.

[7] Beauregard JM, Hofman MS, Pereira JM, Eu P, Hicks RJ (2011). Quantitative (177)Lu SPECT (QSPECT) imaging using a commercially available SPECT/CT system. Cancer Imaging.

[8] Hu J, Seifert R, Karkampouna S (2025). Influence of dosimetry accuracy on the correlation with treatment outcome in a preliminary PSMA radiopharmaceutical therapy study. Eur J Nucl Med Mol Imaging.

[9] Garske U, Sandström M, Johansson S (2012). Minor changes in effective half-life during fractionated 177Lu-octreotate therapy. Acta Oncol.

[10] Willowson KP, Eslick E, Ryu H, Poon A, Bernard EJ, Bailey DL (2018). Feasibility and accuracy of single time point imaging for renal dosimetry following ^177^Lu-DOTATATE ('Lutate’) therapy. EJNMMI Phys.

[11] Heikkonen J, Mäenpää H, Hippeläinen E, Reijonen V, Tenhunen M (2016). Effect of calculation method on kidney dosimetry in ^177^Lu-octreotate treatment. Acta Oncol.

[12] Hänscheid H, Lapa C, Buck AK, Lassmann M, Werner RA (2018). Dose mapping after endoradiotherapy with ^177^Lu-DOTATATE/DOTATOC by a single measurement after 4 days. J Nucl Med.

[13] (2007). The 2007 Recommendations of the International Commission on Radiological Protection. ICRP publication 103. Ann ICRP.

[14] Hosono M, Ikebuchi H, Nakamura Y (2018). Manual on the proper use of lutetium-177-labeled somatostatin analogue (Lu-177-DOTA-TATE) injectable in radionuclide therapy (2nd ed.). Ann Nucl Med.

[15] Le Tourneau C, Lee JJ, Siu LL (2009). Dose escalation methods in phase I cancer clinical trials. J Natl Cancer Inst.

[16] Iwata H, Ishikura S, Murai T (2017). A phase I/II study on stereotactic body radiotherapy with real-time tumor tracking using CyberKnife based on the Monte Carlo algorithm for lung tumors. Int J Clin Oncol.

[17] Hodapp N (2012). Der ICRU-Report 83: Verordnung, Dokumentation und Kommunikation der fluenzmodulierten Photonenstrahlentherapie (IMRT) [Article in German]. Strahlenther Onkol.

[18] Sgouros G, Bolch WE, Chiti A (2021). ICRU REPORT 96, dosimetry-guided radiopharmaceutical therapy. J ICRU.

[19] Emami B, Lyman J, Brown A (1991). Tolerance of normal tissue to therapeutic irradiation. Int J Radiat Oncol Biol Phys.

[20] Hope TA, Abbott A, Colucci K (2019). NANETS/SNMMI procedure standard for somatostatin receptor–based peptide receptor radionuclide therapy with ^177^ Lu-DOTATATE. J Nucl Med.

[21] McDonald AM, Schneider CS, Stahl JM, Oster RA, Popple RA, Mayo CS (2023). A focused review of statistical practices for relating radiation dose-volume exposure and toxicity. Radiat Oncol.

[22] Fukukita H, Suzuki K, Matsumoto K (2014). Japanese guideline for the oncology FDG-PET/CT data acquisition protocol: synopsis of version 2.0. Ann Nucl Med.

[23] Uribe C, Peterson A, Van B (2021). An international study of factors affecting variability of dosimetry calculations, part 1: design and early results of the SNMMI dosimetry challenge. J Nucl Med.

[24] Kobayashi N, Takano S, Ito K (2021). Safety and efficacy of peptide receptor radionuclide therapy with ^177^Lu-DOTA^0^-Tyr^3^-octreotate in combination with amino acid solution infusion in Japanese patients with somatostatin receptor-positive, progressive neuroendocrine tumors. Ann Nucl Med.

[25] Sjögreen Gleisner K, Chouin N, Gabina PM (2022). EANM dosimetry committee recommendations for dosimetry of 177Lu-labelled somatostatin-receptor- and PSMA-targeting ligands. Eur J Nucl Med Mol Imaging.

[26] Editage. www.editage.jp.

